# A Rare Case of Joint Infection due to *Raoultella planticola*

**DOI:** 10.1055/s-0040-1716683

**Published:** 2020-12-14

**Authors:** Kevin Ismair, Yazan Abdeen

**Affiliations:** 1 Arkansas College of Osteopathic Medicine, Fort Smith, AR; 2 Mercy Hospital, Fort Smith, AR

**Keywords:** *Raoultella planticola*, knee infection

## Abstract

*Raoultella planticola* , a gram-negative bacterium, first emerged in late 1900s as *Klebsiella planticola* . It was later classified as *Raoultella* genus in 2001. This nonmotile rod is usually found in soil and aquatic environment. There are two known species of *Raoultella* : *R. planticola* and *R. ornithinolytica* . They are responsible for numerous yet rare infections including cystitis, pneumonia, and bacteremia. To date, only one case of joint or bone infection due to *R. planticola* has been reported. The infection is eradicated after arthroscopic lavage and antibiotic therapy with fluoroquinolones. We present the first case of septic arthritis due to *R. planticola* involving a native knee joint following synovectomy during arthroscopy.

Postoperative knee infections are unusual, but when they occur, these infections should be supervised very closely. Following surgical joint repair, a healthy functioning immune system reacts differently to a prosthetic knee, leaving this artificial body vulnerable for bacteria to adhere to it, thus causing infection at a rapid rate. 1 Postoperative knee infections are commonly caused by *Staphylococcus aureus* and *Streptococcus* species. 2 3 *Raoultella planticola* is an extremely rare pathogen that is associated with knee infection postoperatively, especially in immunocompromised individuals. We present a case of a patient with septic arthritis due to *R. planticola* involving a native knee joint following synovectomy. To our knowledge, this is the first case report of bone or joint infection that occurred due to *R. planticola* in the United States.

## Case Report

A 77-year-old man, with a past medical history of coronary artery disease, diabetes mellitus, osteoarthritis, hypertension, obesity, and right knee arthroplasty with a complication of an infected prosthetic knee joint 7 years before presentation, was evaluated by his primary care provider for left knee pain due to septic arthritis. The patient presented with fever, limited range of motion, swelling, and pain in his left knee joint. A referral to the Orthopedic Surgery Department resulted in a removal of infected left total knee arthroplasty with placement of articulating antibiotic spacer. Intravenous vancomycin (1,500 mg) was administered for 6 weeks, and the patient was scheduled for a follow-up visit with his orthopedic surgeon following the treatment. Upon reexamination, the patient's wound healed; however, an aspiration of the left knee was unsuccessful due to the absence of drainage. The patient was treated with vancomycin for two weeks.

**Fig. 1:**
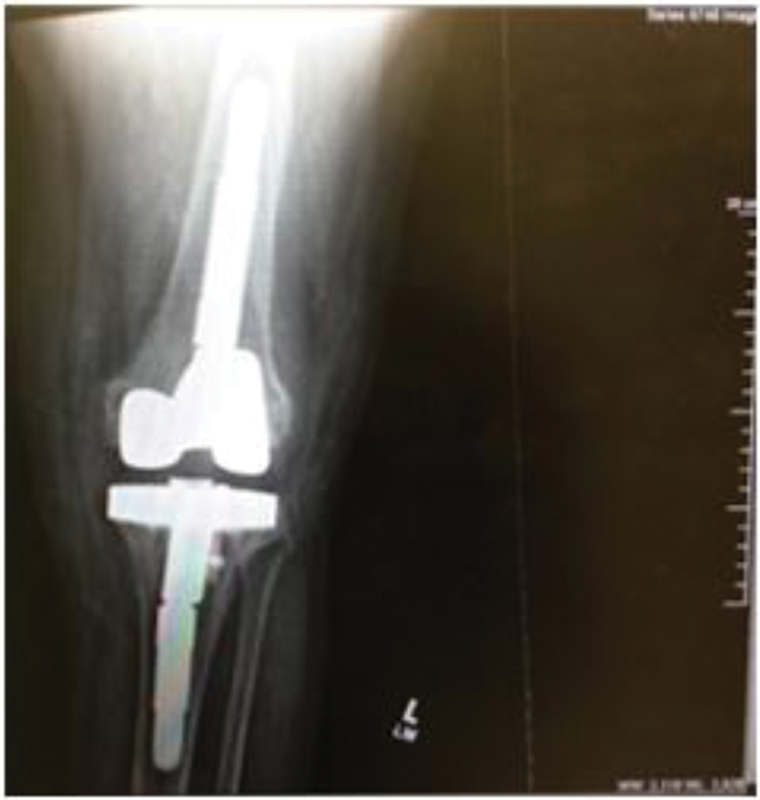
X-ray: lateral view of the insertion of a spacer postsurgery of the left knee.

**Fig. 2:**
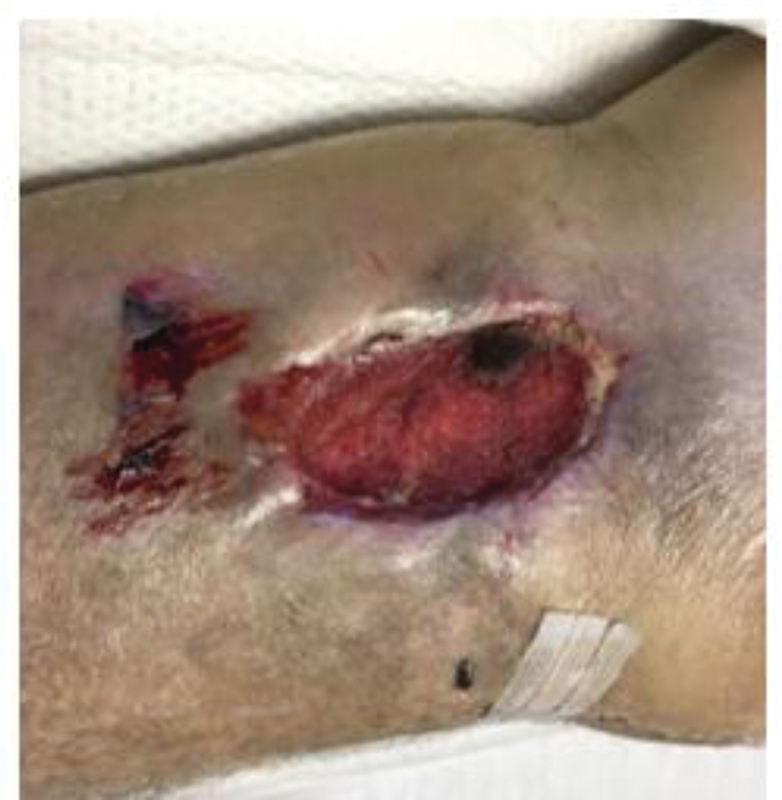
Open wound after left knee arthroplasty.

Following the two weeks of treatment, the patient presented at the hospital for an orthopedic evaluation with significant lymphedema and drainage from the left leg. The patient underwent a stage 1 knee spacer procedure. A blood culture with gram staining was ordered, and results confirmed an aerobic growth of 1 +  or few *R. planticola.* The patient was admitted for inpatient care. One week later, the patient presented with fever, severe pain, limited range of motion, and stiffness. Due to a concern of a suspected *R. planticola* prosthetic left knee infection, a left knee arthroplasty revision stage 1 procedure was performed ( Fig. 1 ). On day 1 postoperatively, the patient was placed on a vacuum-assisted wound closure therapy on the upper part of the left lower leg, during which his wound was drained ( Fig. 2 ). Physical examination revealed that the patient had poor knee mobility, arthralgia, and dyspnea. High-dose intravenous levofloxacin (750 mg/150 mL) every 24 hours for 6 weeks was administered. Additionally, the patient was given rivaroxaban for deep vein thrombosis prophylaxis and was placed in long-term acute care.

## Discussion

*Raoultella planticola* , an aerobic gram-negative bacterium, first emerged in the late 1900s as *Klebsiella planticola* and was later renamed *R. planticola* in 2001. There are two known species of *Raoultella* , *R. planticola* and *R. ornithinolytica* , that have been shown to be responsible for numerous infections including cystitis, pneumonia, and bacteremia. *Raoultella planticola* is found in environments containing soil, plants, and water. It is a potentially fatal opportunistic bacterium that has been rarely involved in clinical infections. Enteric translocation and invasive medical procedures are the main potential sources of infection caused by this organism. 4 5 6 7

The mechanism underlying the mode of action of *R. planticola* is not clearly understood. However, several reports have indicated that *R. planticola* is commonly associated with gastrointestinal, urinary tract, and joint infections. 4 5 7 Bonnet et al reported the first definitive case of a joint infection due to *R. planticola* following synovectomy and intra-articular injection of corticosteroid during arthroscopy. Interestingly, *R. planticola* showed antibiotic susceptibility to β-lactams and all other additional antibiotics tested. 4 Results were confirmed through synovial fluid collection and mass spectrometry, whereas the results pertaining to our case were validated by means of blood cultures, laboratory tests, and acid-fast bacteria staining.

Risk factors for this infection include immunocompromised status, obesity, diabetes, rheumatoid arthritis, smoking history, prior surgeries, or prior administration ofcorticosteroids. 1 3 4 5 7 8 9 Clinical presentation often involves fatigue, redness and swelling, wound drainage, and increased pain with limited mobility. 1

Notably, to our knowledge, our case is the only report of joint infection due to *R. planticola* involving a knee joint postarthroscopy. Based on the extensive medical history, including several arthroplasties and diabetes, the patient is at a greater risk for the development of infection.

A series of tests, including a complete blood count, C-reactive protein, erythrocyte sedimentation rate, and blood cultures, were performed to confirm the responsible pathogen and susceptibility to spectrum of antibiotics in this case. Evaluation of blood cultures indicated that *R. planticola* was susceptible to a variety of antibiotics, specifically cefepime, ertapenem, levofloxacin, meropenem, and piperacillin-tazobactam. Nevertheless, *R. planticola* was found to be resistant to β-lactams, ampicillin, sulbactam, cefazolin, ceftriaxone, gentamicin, and trimethoprim-sulfamethoxazole.

Treatment of joint infections due to *R. planticola* often requires antimicrobial therapy, specifically administration of fluoroquinolones. 4 Similarly, in our case, levofloxacin, a fluoroquinolone, was administered for 6 weeks. While it remains unclear as to why the number of human *R. planticola* infections is on the rise, it is important to consider this environmental bacterium as part of the differential diagnosis of joint infection, especially in patients with impaired immune system and significant comorbid conditions.
